# Responses of tree defoliators to traffic-derived particulate matter and trace elements along a roadside pollution gradient

**DOI:** 10.1038/s41598-026-41296-7

**Published:** 2026-02-21

**Authors:** Hanna Moniuszko, Robert Popek, Arkadiusz Przybysz, Adrian Łukowski

**Affiliations:** 1 Centre for Climate Research SGGW, Warsaw University of Life Sciences—SGGW (WULS— SGGW), Nowoursynowska 166, Warsaw, 02-787 Poland; 2 Faculty of Forestry and Wood Technology, Poznań University of Life Sciences, Wojska Polskiego 71e, Poznań, 60-625 Poland

**Keywords:** Air pollution, Environmental stress, Herbivory, Phytoremediation, Road verges, Ecology, Ecology, Environmental sciences

## Abstract

Traffic-derived particulate matter (PM) and trace elements (TEs) are pervasive stressors in roadside habitats. Although some insects, including pest species, appear to thrive despite roadside pollution, such observations may risk overgeneralization. We examined the responses of *Yponomeuta padella*, as a model roadside defoliator, to PM and TE pollution by rearing larvae on two hosts—*Crataegus monogyna* and *Prunus cerasifera*—obtained along a real-world pollution gradient (control, sidewalk, roadside). Leaves were characterized for physical traits (SLA, toughness), PM load, and TE concentrations. PM pollution followed a consistent control < sidewalk < roadside pattern across categories and size fractions; Cu, Fe and Sr showed the same spatial trend, while As, Cd, and Pb were absent in most control samples. Choice tests revealed strong larval avoidance of contaminated foliage (roadside and sidewalk) irrespectively of host species. Emergence dynamics (logistic models) showed slower growth rates and later inflection points with increasing pollution, and eclosion success declined significantly from control (90.1%) to sidewalk (82.5%) and roadside (77.1%). Adult body mass was lowest for roadside diets. Host species differed in SLA, toughness, and several accumulation metrics, but site effects dominated developmental outcomes. The results demonstrate context-dependent susceptibility of moths to PM and link adult performance costs to larval diets on PM-contaminated foliage. We propose that co-occurring pollution and stress typical of road verges act as an ecological filter shaping insect survival, with implications for habitat degradation in roadside ecosystems.

**Supplementary Information:**

The online version contains supplementary material available at 10.1038/s41598-026-41296-7.

## Introduction

Airborne particulate matter (PM), a significant component of urban and roadside air pollution, poses a non-decreasing threat to both environmental and human health^1,2^. Traffic-derived PM consists of a complex mixture of organic and inorganic substances, with toxic trace elements (TEs) and polyaromatic hydrocarbons (PAHs) being the most significant contaminants, which are inhaled by humans and other vertebrates or settle on invertebrates’ bodies^2–5^. Leaves and stems of roadside vegetation of different types (trees, shrubs, and meadows) act as passive collectors of airborne PM, thus contributing to the partial removal of the above-mentioned toxins from the air^4,6,7^. In addition, toxicants that do not settle on vegetation may deposit onto the soil surface, migrate downward, and be absorbed by plant roots, eventually reaching vegetative organs^8^. Thus, PM deposition should not only be considered as a toxicological burden, but also as an abiotic factor that may restructure interactions across trophic levels.

While phytoremediation is a beneficial ecosystem service, recent studies on the phyllosphere-associated arthropods indicate that ecological consequences of the phyto-filtration of air exceed the plant–pollutant system^9,10^. Among the less explored yet ecologically significant impacts of phytoremediation from airborne PM is the hidden costs of this phenomenon paid by herbivorous and even predatory arthropods. Having settled on vegetation, PM and PM-associated PAHs and TEs remain on leaf surfaces, are bound by epicuticular waxes or may even migrate deep into foliar tissues^4,11–13^. Subsequently, these chemicals can be ingested by phytophagous insects and mites during the foraging on plant tissues and sap reducing their biomass and population dynamics^9,14,15^, or the contaminant can mechanically prevent micro-predators from occupation of shelters located within leafy veins and trichomes^10^. Moreover, lumps of PM can adhere to the mites’ bodies making it impossible to be avoided even by relatively mobile species^10^. This mechanical aspect distinguishes PM from gaseous pollutants or soil-derived metals, positioning it as a multifaceted stressor in ecological systems.

Despite these challenges, numerous insect pests are often reported as relatively tolerant to polluted environments, including those impacted by road traffic, contrary to beneficial invertebrates^10,16–21^. This generalization, however, may overlook hidden behavioural and demographic responses to pollution in taxa associated with non-cultivated plants under real-life PM pollution such as that along roadsides^19,22^. Arthropods with narrow host ranges and low flexibility in food selection may be particularly vulnerable when their host plants grow in polluted environments, lacking the capacity to avoid contaminated resources.

Among models used to investigate plant–insect–environment interactions, moths of the genus *Yponomeuta* (Lepidoptera: Yponomeutidae), particularly monophagous *Y. evonymellus* (L.), have been frequently studied^23–25^. Another species, oligophagous *Y. padella* L. (orchard ermine), is especially well suited for research on degraded environments. As its larval host plants (*Crataegus* spp. and *Prunus* spp.) are commonly found in urban and roadside plantings, the orchard ermine provides a promising model for examining the further peculiarities of the effects of foliar PM deposition on insect herbivores in urban and peri-urban environments. The fixed oviposition site selected by the female moth combined with restricted mobility of caterpillars, impose a narrow environmental window for larval development^24^. This increases larval susceptibility to local habitat conditions, including the chemical quality of food resources. Thus, *Y. padella* provides a tractable model for testing broader predictions about the sensitivity of specialized folivores to particulate pollution.

In this study, we examine the influence of real-life PM concentrations on foraging preferences as well as on food quality and larval development of *Y. padella* fed on two species of its host plants, frequently planted as roadside greenery. By doing so, we test the assumption of chewing herbivore tolerance to pollution. Specifically, we assessed: (i) larval feeding preferences when exposed to foliage of *C. monogyna* or *P. cerasifera* obtained from sites with increasing concentrations of traffic-associated PM; (ii) larval survival rates, adult emergence dynamics, and body mass of moths developed from caterpillars kept on foliage contaminated with real-life PM concentrations. It is hypothesized that (i) when given a choice, larvae will avoid contaminated foliage. This behaviour may be evident under laboratory conditions, where larvae can freely choose between contaminated and uncontaminated food sources. In contrast, in natural environments, larvae are usually restricted to the tissues on which their eggs were laid, which limits their ability to avoid contamination. Therefore, an additional hypothesis is that (ii) in the field, larvae may be unable to escape contaminated foliage due to the limited availability of clean resources, which in turn increases their exposure and susceptibility to the detrimental effects of PM, ultimately impairing their development and fitness.

## Results

### Physicochemical leaf traits of the host plants

Significant differences in the physical parameters of foliage between *C. monogyna* and *P. cerasifera* were found. Mean SLA was significantly lower in *C. monogyna* (169.47 ± 1.68 cm²/g) than in *P. cerasifera* (190.01 ± 2.30 cm²/g), with host plant species accounting for a substantial portion of the variation (F = 55.9843, *p* < 0.0001, R² = 0.2245). Similarly, mean leaf toughness differed significantly between the two species: *C. monogyna* leaves were less tough (146.96 ± 1.32 gf/mm²) compared to those of *P. cerasifera* (156.99 ± 1.22 gf/mm²; F = 27.953, *p* < 0.0001, R² = 0.2401).

In contrast, the effect of site (roadside, sidewalk, control) on both mean SLA and toughness was not statistically significant (SLA: F = 1.6180, *p* = 0.2078; toughness: F = 0.1248, *p*= 0.8829; *n*=90 for each trait, 30 leaves per site variant). Similarly, the interaction between host plant and site had no significant influence on either parameter (SLA: F = 2.6636, *p* = 0.0788; toughness: F = 1.3411, *p* = 0.2701; *n*=90).

*Crataegus monogyna* and *P. cerasifera* also differed significantly in terms of accumulation of all PM categories and size fractions examined, except for _W_PM and PM_2.5–10.5_. Roadside, sidewalk and control foliage differed significantly in concentrations of PM of all categories and size fractions, while host plant and site as combined factors—only in terms of total PM, _W_PM and PM_2.5–10.5_ (Table 1).

**Table 1:** Variance analysis of results for the concentration of PM measured on host plants (*C. monogyna* and *P. cerasifera*) and sites (roadside, sidewalk and control).

PM accumulation among the examined host plants												
	Total PM (µg/cm^2^)		sPM (µg/cm^2^)		wPM (µg/cm^2^)		PM size fraction (µg/cm^2^)					
							PM_10–100_		PM_2.5–10.5_		PM_0.2–2.5.2.5_	
R^2^	0.9973		0.9952		0.9955		0.8753		0.9961		0.9901	
ANOVA	F	p	F	p	F	p	F	p	F	p	F	p
Host plant	6.9082	0.0111	11.243	0.0015	0.0589	0.8091	51.018	< 0.0001	0.7826	0.3803	38.845	< 0.0001
Site	399.37	< 0.0001	273.35	< 0.0001	278.85	< 0.0001	297.18	< 0.0001	274.01	< 0.0001	407.32	< 0.0001
Host plant × site	3.2311	0.0473	0.6267	0.5382	7.7257	0.0011	2.2663	0.1135	5.9617	0.0046	0.5826	0.5619
N	60		60		60		60		60		60	

**Fig. 1:**
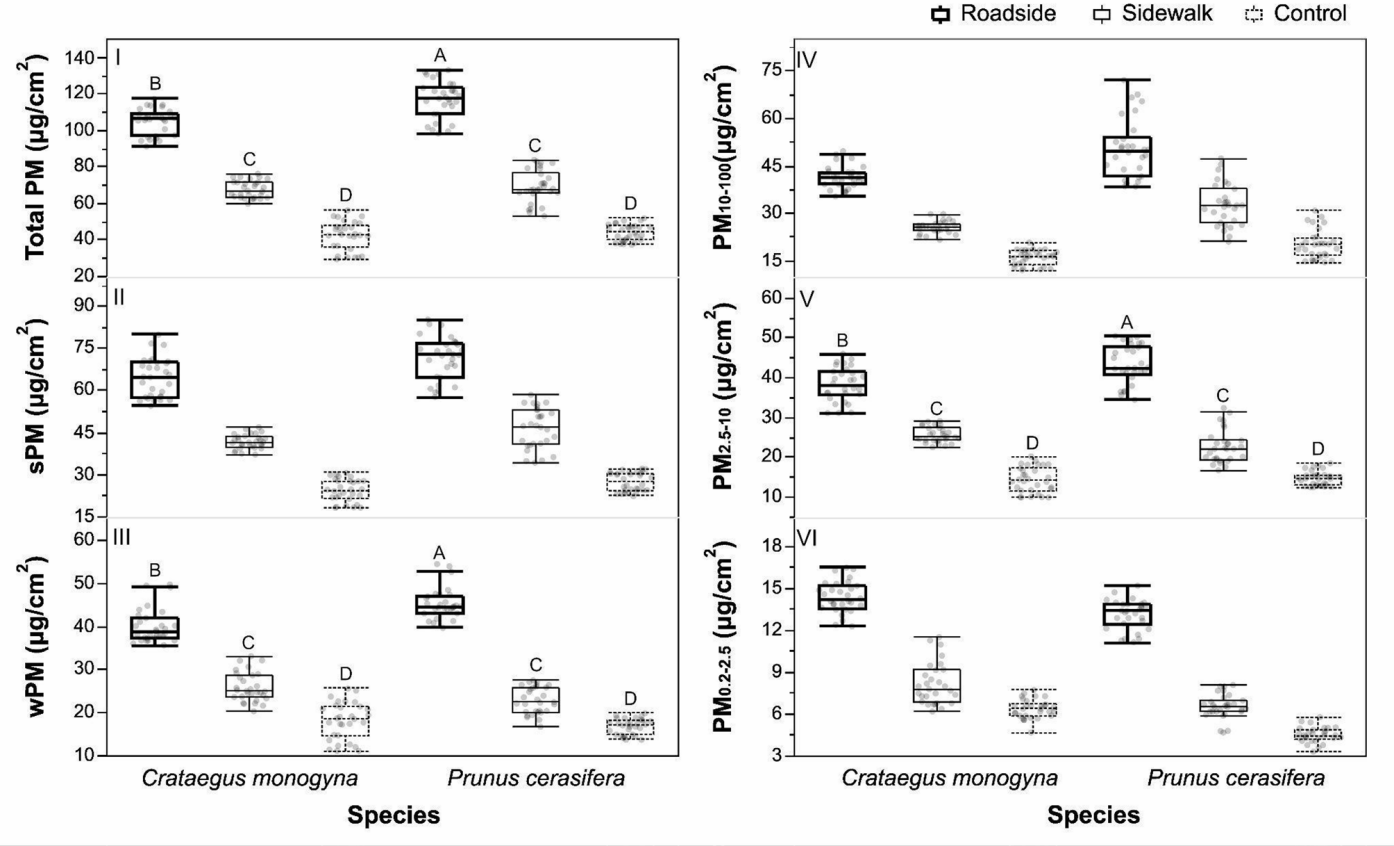
Particulate matter accumulation by *C. monogyna* and *P. cerasifera* from three study sites (roadside, sidewalk and control). Capital letters indicate significant differences in interaction between host plant × site.

Leaves harvested from the roadside, sidewalk and control sites were characterized by decreasing concentrations of every PM category and size fraction, irrespectively of the host plant species (Fig. 1).

Among detected TEs, concentrations of only Cu and Fe did not differ significantly between *C. monogyna* and *P. cerasifera* (Table 2).

**Table 2:** Variance analysis of results for TEs content in leaves measured on host plants (*C. monogyna* and *P. cerasifera*) and sites (roadside, sidewalk and control).

Trace elements content in leaves of the examined host plants																		
	Ba (mg/kg)		Cu (mg/kg)		Fe (mg/kg)		Mn (mg/kg)		Mo (mg/kg)		Pt (mg/kg)		Rb (mg/kg)		Sr (mg/kg)		Zn (mg/kg)	
R^2^	0.8729		0.9392		0.9942		0.8851		0.4678		0.3599		0.9823		0.9612		0.9750	
ANOVA	F	p	F	p	F	p	F	p	F	p	F	p	F	p	F	p	F	p
Host plant	53.192	< 0.0001	3.4898	0.0672	0.0053	0.9424	61.067	< 0.0001	5.0462	0.0288	10.599	0.0020	3.5130	0.0663	124.25	< 0.0001	145.41	< 0.0001
Site	2.7563	0.0725	87.134	< 0.0001	76.463	< 0.0001	14.478	< 0.0001	0.2317	0.7940	7.0555	0.0019	124.82	< 0.0001	42.628	< 0.0001	2.9572	0.0604
Host plant × site	0.4218	0.6580	8.3103	0.0007	4.6804	0.0134	28.315	< 0.0001	0.4296	0.6530	4.5694	0.0147	11.633	< 0.0001	0.0346	0.7091	6.7033	0.0025

**Fig. 2:**
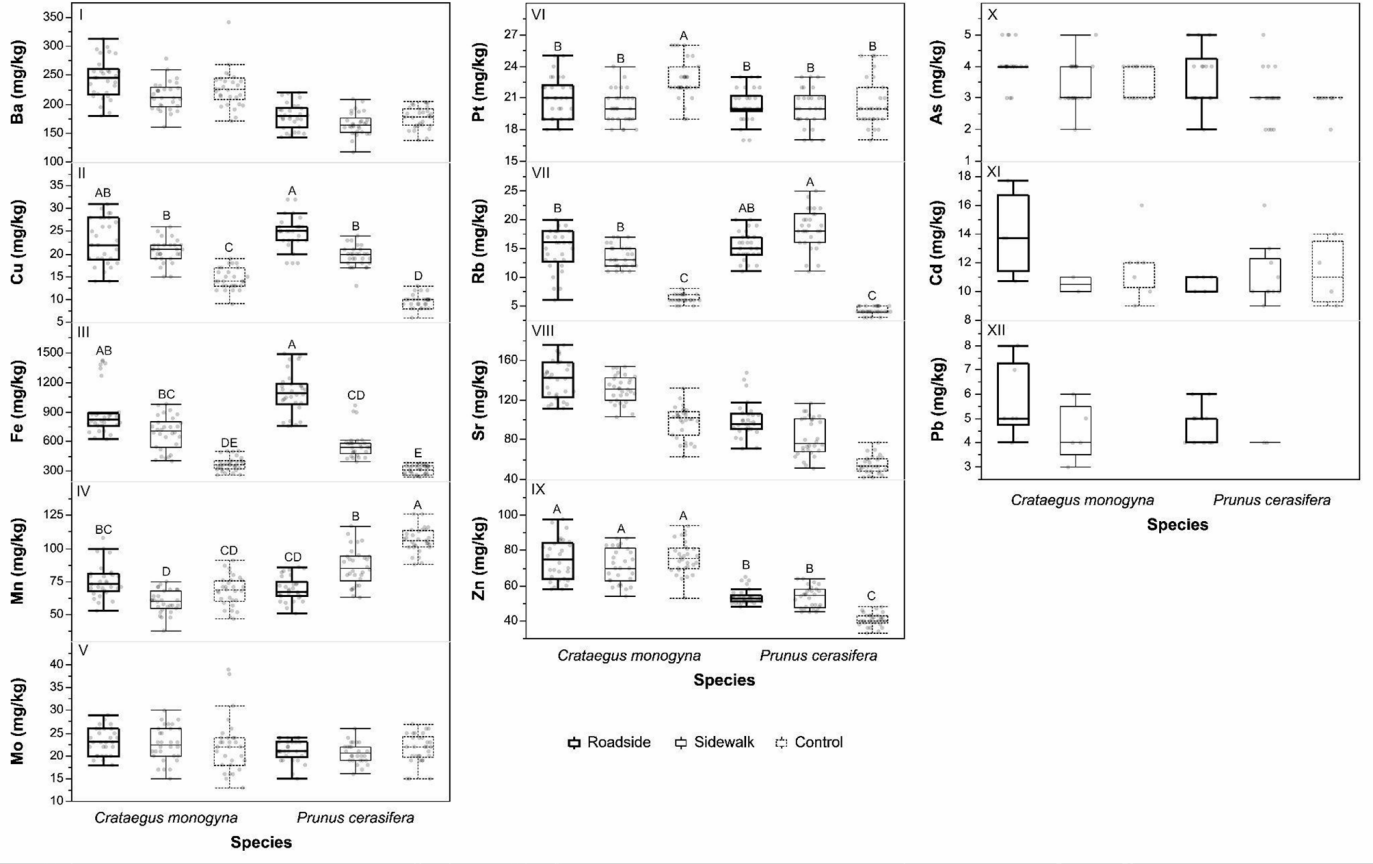
Concentrations of selected TEs in leaves of *C. monogyna* and *P. cerasifera* collected from three sites differing in traffic-related pollution. Capital letters indicate significant differences in interaction between host plant × site.

Roadside leaves exhibited the highest concentrations of Cu, Fe, and Sn. Other TEs, Ba, Mn, Mo, Pt, Zn, and Rb, exhibited no clear spatial distribution pattern (Fig. 2).

### Feeding preference and performance of *Y. padella*

**Fig. 3:**
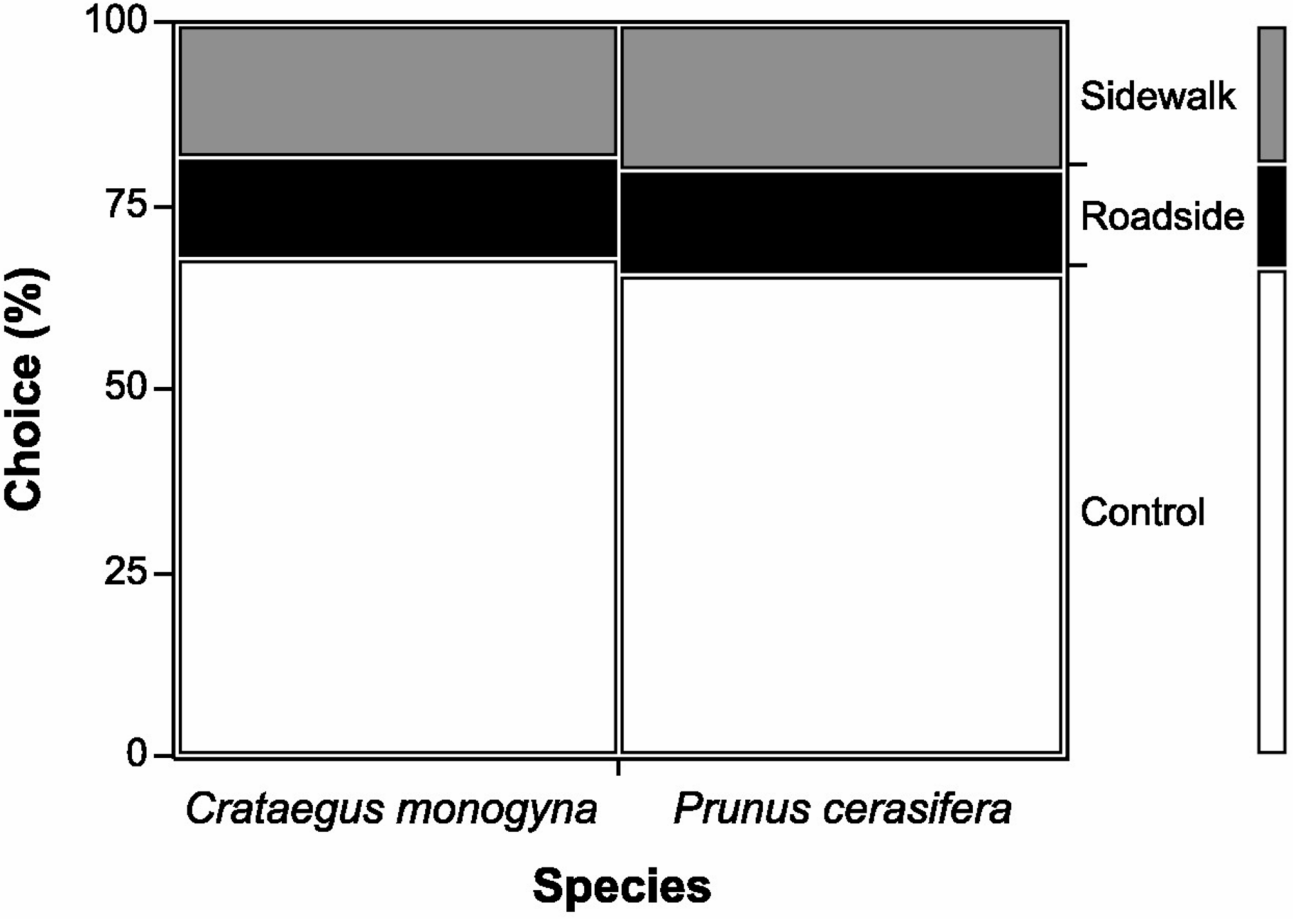
Shares of *C. monogyna* and *P. cerasifera* leaves chosen from different sites by *Y. padella* larvae (*n* = 50 larvae per plant species).

The analysis of food preferences showed a clear deviation from a random distribution for both host plants studied (Fig. 3). In *P. cerasifera*, a strong preference was observed for the control leaves (66% of choices), while leaves from roadside and sidewalk areas were chosen much less frequently (14% and 20% of choices, respectively; χ^2^ = 24.28; *p*< 0.001; *n* = 50 larvae). A similar pattern of preference was observed in *C. monogyna*, where the control leaves were also chosen most frequently (68% of choices), and leaves from roadside and sidewalk areas accounted for 14% and 18% of choices, respectively (χ^2^ = 27.16; *p*< 0.001; *n* = 50 larvae). The chi-square test of independence showed no significant differences in preferences between species (χ^2^ = 0.068; *p*= 0.967), suggesting that the pattern of leaf choice was similar for both species studied.

For *C. monogyna*, individuals feeding on roadside leaves exhibited a slower growth rate and a later inflection point than those from the sidewalk. Similarly, for *P. cerasifera*, growth was slower and emergence occurred later in the roadside variant compared to the sidewalk. Notably, irrespectively of the host plant, the fastest development and earliest inflection point occurred in the control groups (Table 3).

**Table 3:** Means (± SE) and variance analysis of the parameters of the logistic model describing emergence dynamics (growth rate and inflection point) of *Y. padella* kept on *C. monogyna* and *P. cerasifera* foliage from three experimental sites (roadside, sidewalk or control).

Parameters of the logistic model describing emergence dynamics						
			Growth rate (1/day)		Inflection point (day)	
Host plant	Site variant	*n*	Mean (± SE)		Mean (± SE)	
*Crataegus monogyna*	Roadside	10	0.979 (± 0.044)		7.283 (± 0.156)	
	Sidewalk	10	1.083 (± 0.046)		6.153 (± 0.154)	
	Control	10	1.455 (± 0.197)		3.933 (± 0.241)	
*Prunus cerasifera*	Roadside	10	1.149 (± 0.066)		7.214 (± 0.233)	
	Sidewalk	10	1.125 (± 0.051)		6.192 (± 0.097)	
	Control	10	1.131 (± 0.067)		4.158 (± 0.245)	
R^2^			0.2071		0.8373	
ANOVA			F	p	F	p
Host plant			0.2311	0.6327	0.1659	0.6854
Site			3.3043	0.0443	138.61	< 0.0001
Host plant × site			3.6303	0.0332	0.2860	0.7524
N			60		60	

**Fig. 4:**
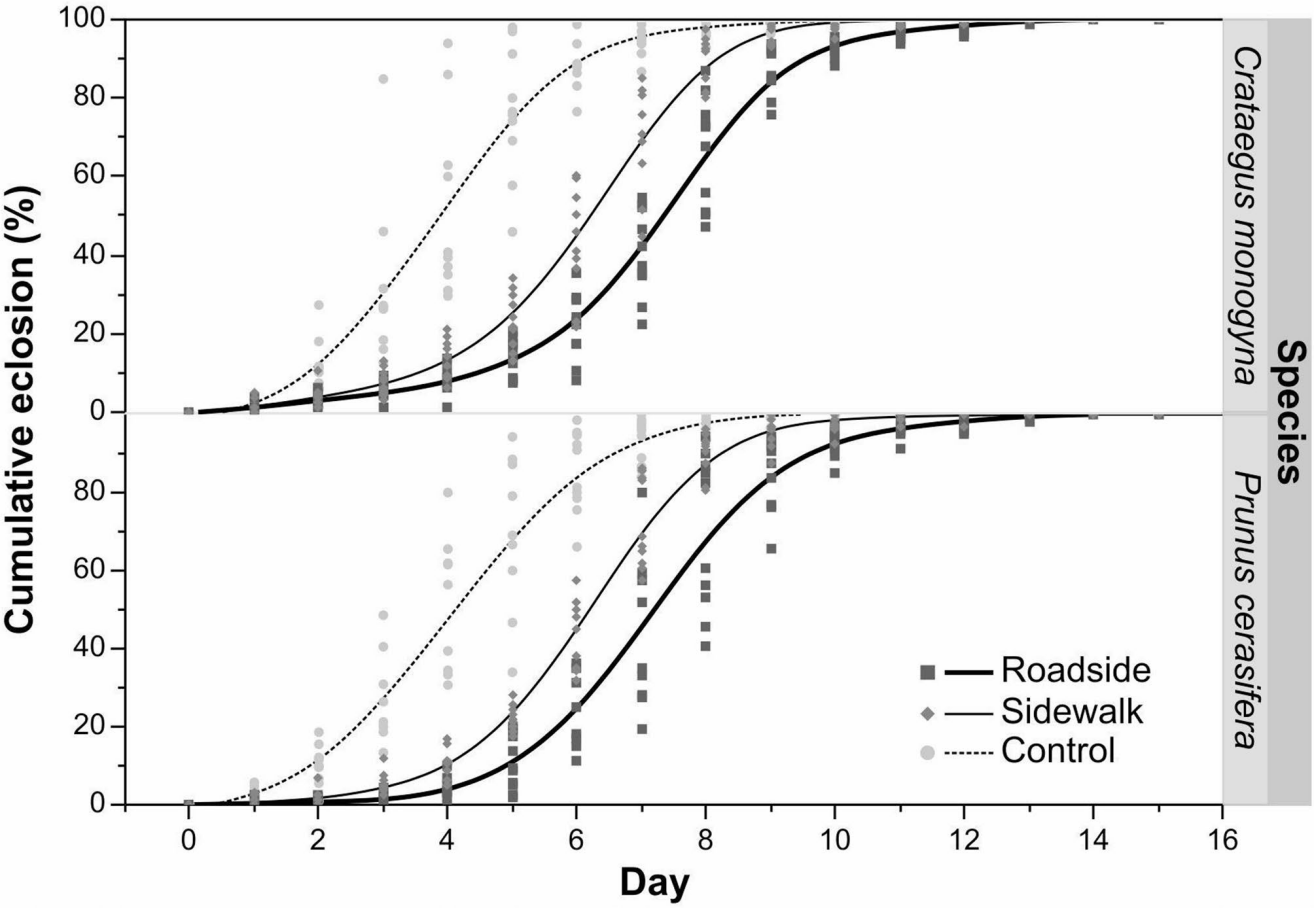
Cumulative eclosion curves (%) of *Y. padella* reared on *C. monogyna* (*n* = 10 nests per site variant) and *P. cerasifera* foliage collected from three experimental sites.

Figure 4 illustrates the delayed emergence of *Y. padella* imagines under increased PM pollution.

**Fig. 5:**
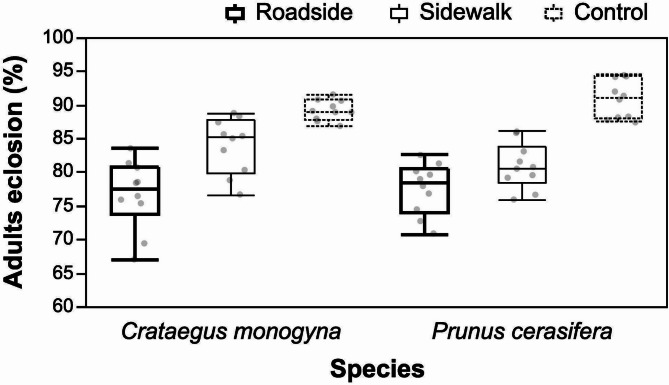
The percentage of adult eclosion from *Y. padella* pupae fed on *C. monogyna* and *P. cerasifera* leaves harvested from three experimental sites.

Eclosion success of *Y. padella* decreased with increased pollution gradient (Fig. 5). A two-way ANOVA revealed a strong effect of collection site (site) on the percentage of eclosion (F = 70.69, *p* < 0.0001), while neither species identity (F = 0.0017, *p* = 0.9671) nor the interaction between species and site (F = 2.96, *p* = 0.0605) reached statistical significance. The coefficient of determination for the model (R² = 0.73) indicates that a substantial proportion of the variability in eclosion rates is explained by the experimental factors. Mean eclosion rates were highest for leaves collected from the control site (90.14% ±0.53), followed by the sidewalk (82.50% ±0.90), and lowest at the roadside (77.13% ±0.99). This pattern was consistent across both *C. monogyna* and *P. cerasifera*.

**Fig. 6:**
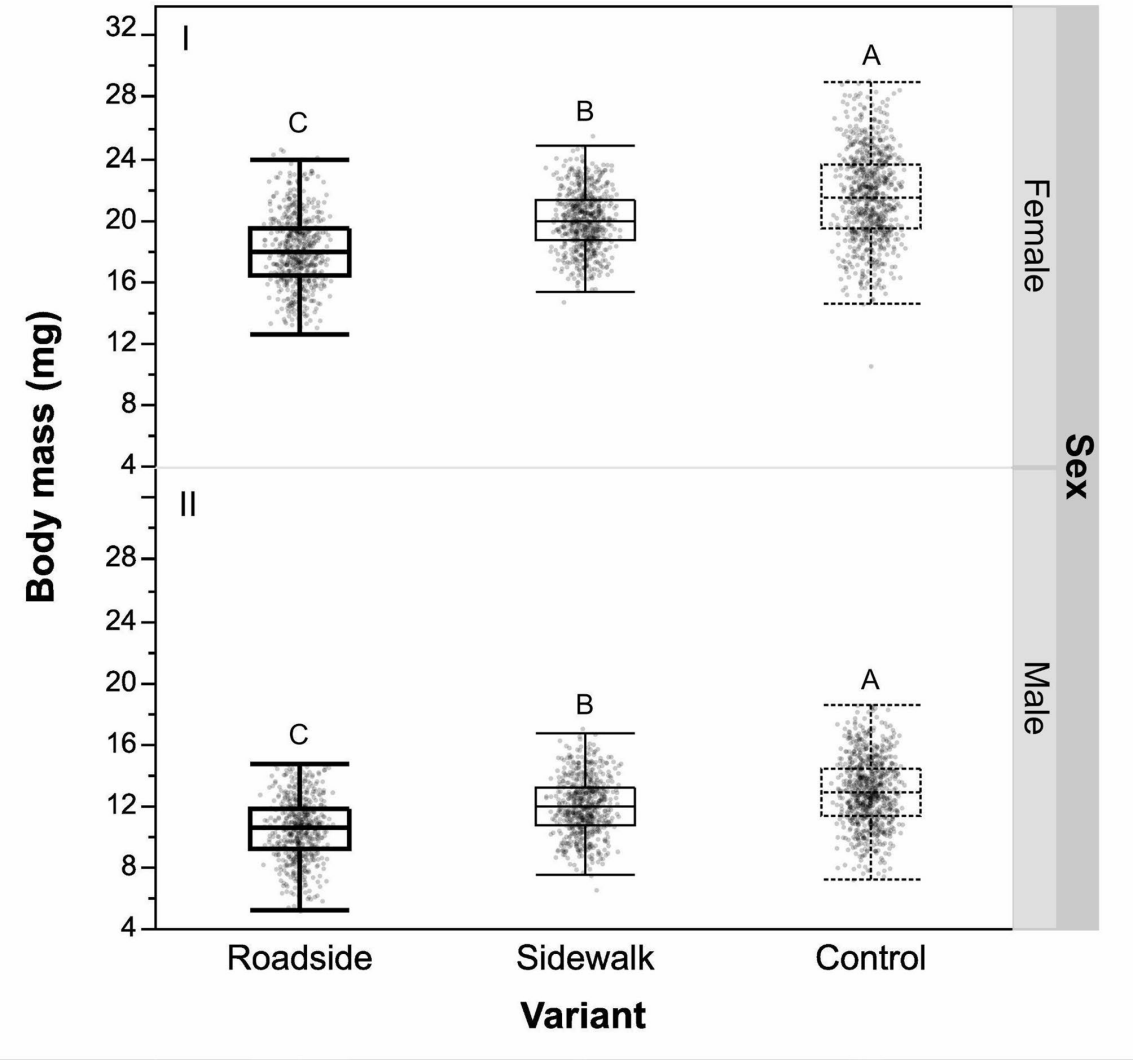
Final adult masses gained by individuals reared on the foliage of *P. cerasifera* and *C. monogyna* collected in three experimental sites.

For both *C. monogyna* and *P. cerasifera*, the lowest mean masses of adult moths were recorded in the roadside variant (Fig. 6).

ANOVA results confirmed a significant effect of pollution levels characterising three experimental sites on adult mass for both females (*F* = 41.678, *p* < 0.001) and males (*F* = 265.484, *p* < 0.001), whereas host plant species and the interaction between factors were non-significant (Table 4).

**Table 4:** Means (± SE) and variance analysis of masses of males and females of *Y. padella* reared from larvae fed on *C. monogyna* and *P. cerasifera* foliage harvested in three experimental sites.

Masses of adult insects					
Host plant	Site variant	Female mass (mg)		Male mass (mg)	
		*n*	Mean (± SE)	*n*	Mean (± SE)
*Crataegus monogyna*	Roadside	280	18.05 (± 0.13)	284	10.41 (± 0.11)
	Sidewalk	345	20.04 (± 0.10)	333	12.02 (± 0.10)
	Control	373	21.45 (± 0.15)	369	12.94 (± 0.12)
*Prunus cerasifera*	Roadside	320	18.03 (± 0.13)	299	11.59 (± 0.12)
	Sidewalk	304	19.92 (± 0.11)	303	11.97 (± 0.11)
	Control	425	21.63 (± 0.14)	434	12.94 (± 0.10)
R^2^		0.2494		0.1949	
ANOVA		F	p	F	p
Host plant		0.0707	0.7915	0.2068	0.6520
Site		416.78	< 0.0001	268.44	< 0.0001
Host plant × site		0.9135	0.4081	0.6258	0.5406
N		2047		2022	

## Discussion

### Traits of roadside greenery and pollution distribution

*Crataegus monogyna* and *P. cerasifera* differed significantly in several parameters: (i) SLA and leaf toughness, (ii) total PM accumulation capacity—both higher in *P. cerasifera*, and (iii) concentrations of most detected TEs, which varied in direction between the two species. These differences can be attributed to species-specific traits of hosts. Interspecific variation in leaf physical properties as well as in PM and TE accumulation patterns is commonly observed in plants^26–28^. Although leaf toughness is considered a good predictor of herbivory levels^27^, and thus might be expected to influence larval performance, such host-related effects appear secondary in light of the below-discussed results regarding PM pollution across experimental sites and the outcomes of biological assays.

Regardless of plant species, PM concentrations consistently followed the control < sidewalk < roadside gradient, validating the site setup as a reliable proxy for environmental PM load. A similar decreasing pattern of PM deposition from the roadside to sidewalk was also observed in earth berms covered by herbal plants and shrubs, as well as on climber-covered walls and single trees. These findings confirm both the severity of roadside conditions and the mitigating effect of green infrastructure in reducing traffic-derived PM along transportation corridors^5,29^. Furthermore, recent studies have demonstrated a negative correlation between PM accumulation on roadside vegetation and invertebrate biodiversity indices, highlighting the ecological cost of foliar pollutant retention in such habitats^29^.

Among TEs, only Cu, Fe and Sr followed the control < sidewalk < roadside gradient. This can be attributed to their widespread use in vehicle components and their gradual release through abrasion and corrosion^30^. Concentrations of the remaining TEs were characterized by a non-uniform pattern, which could be attributed to their differentiated abundance in the environment as well as to physical and physiological peculiarities of their uptake by plants. For instance, Ba is known for its ubiquity^31,32^ while Mo and Zn are micronutrients^33,34^. The observed Mn concentrations were relatively low, and the interspecific differences were small, suggesting limited ecological relevance. We interpret the slightly higher Mn content as a reflection of more intensive metabolism and photosynthetic activity in plants growing at the control site, farther from the road. Similar differences were observed in other deciduous species growing at the trafficked roadside and municipal parks, with the latter reaching up to 700 ppm of Mn in foliage tissues and fluctuating in time^35^. In addition, *P. cerasifera* is known for efficient uptake and translocation of micronutrients to green tissues; as such, it has been used as a rootstock to enhance micronutrient acquisition under alkaline soil conditions^36^.The presence of Rb can be attributed to its occurrence in parent rock, from which it migrates into soil and may be resuspended as dust or absorbed by plants due to its chemical similarity to K^37,38^. Additionally, Rb has been identified as an emerging pollutant in sintering dust from the steel industry^39^. Arsenic, Cd, and Pb known for their toxicity were detected on the roadside and sidewalk where their presence is not uncommon^5,9,40^, while these TEs did not occur on the control side or were detected in single samples only. Moreover, vegetation adjacent to highways also accumulates low, medium and heavy molecular weight PAHs, including toxic benzo(a)pyrene^5^. This only enhances the effect of high PM concentrations detected on the traffic-exposed site and explains the results of the larval choice test and further consequences of PM exposure on *Y. padella* development.

### Larval feeding preferences

The fauna’s responses to environmental pollutants generally involve repulsion-like and attraction-like strategies as well as behavioural shifts which can affect it on the individual, community, population and evolutionary levels^41^. In the case of *Y. padella*, for both host species, larvae strongly preferred pollution-free foliage. This pattern indicates a capability to discriminate leaf quality related to pollution exposure. However, the habitat-source effects in the field reflect environmental constraints (related to low mobility and foraging mode) rather than larval choice, while the observed laboratory preference represents a plastic response under controlled conditions. In contrast, the caterpillars of western tussock moth, *Orgyia vetusta*, (Lepidoptera: Lymantriidae) when given a choice, most frequently selected the foliage of *Quercus lobata* from highway-adjacent trees in California, USA^20^. The densities of *Eriocrania* miners (Lepidoptera: Eriocraniidae) foraging on *Betula* leaves growing around a facility emitting SO_2_ and TEs in Finland revealed preference for moderately polluted foliage^17^. A certain level of immunity to gaseous and TE air pollution was also observed in Danish populations of leaf-mining caterpillars of Microlepidoptera, as opposed to other groups of butterflies^18^. The response of *Helicoverpa armigera* (Lepidoptera: Noctuidae) larvae to coal-derived PM varied with developmental stage—while neonates actively avoided PM-contaminated feeding sites, older instars exhibited no such aversion^15^. This also contrasts with our findings, as in the present research final-instar *Y. padella* larvae were used. It should be noted, however, that coal-derived PM differs in composition from traffic-related PM, although both contain toxic TEs. Nonetheless, our results emphasize that even within generally pollution-tolerant groups such as moths, individual traits and environmental context can markedly shape behavioural strategies.

### The impact of PM on *Y. padella* development dynamics

Pollution intensity exerted strong effects on eclosion dynamics and success. On both host plant species, the control < sidewalk < roadside gradient remained clear, with the fastest development and highest adult emergence success observed on control leaves. These results are consistent with laboratory experiments on non-moth butterflies. *Heliconius ethilla* (Lepidoptera: Nymphalidae) fed with *Passiflora edulis* leaves treated with sedimentable PM, similarly to *Y. padella*, showed reduced body mass and size^42^. Another nymphalid, *Bicyclus anynana*, also exhibited altered development time and lower pupal mass as a result of feeding on vegetation exposed to artificially generated haze^43^.

The effects discussed may result from direct toxicity of contaminated food, which impacts insects at the molecular level. Piccini et al.^44^ revealed that *Pieris brassicae* (Lepidoptera: Pieridae) butterflies reared on cabbage treated with PM extracts—designed to mimic pollution gradients in urban and suburban areas—exhibited genotoxic effects involving micronucleus abnormalities. Moreover, pollution intensity was reflected in the extent of DNA damage. It cannot be excluded that similar yet-to-be-identified mechanisms are involved in the physiology of *Y. padella* inhibiting roadside greenery. The more that its response to PM exposure appears more similar to that of non-moth butterflies than to highly tolerant moth species. An additional factor contributing to the reduced performance of chewing insects on roadside greenery is environmental stress. In general, road verges provide an important habitat for a variety of lepidopteran species^45^. However, an analysis by Koricheva et al.^46^ indicates that feeding on experimentally stressed plants can reduce the performance of chewers. Together, these findings suggest that the combined impact of toxic pollutants and stress typical of roadside habitats creates an ecological filter that shapes insect survival. It also highlights that PM pollution should be viewed not only as an air-quality issue but also as a driver of habitat decline for herbivorous insects associated with communication routes.

## Conclusions

Overall, our findings demonstrate that in roadside environments, where particulate and TE pollution is a key driver of habitat degradation, moths exhibit context-dependent susceptibility to PM. Larval avoidance of contaminated foliage, observed even in final-instar *Y. padella*, indicates that assumptions of general herbivore tolerance to pollution are oversimplified, reflecting the importance of species identity, feeding guild (defoliating, mining or shelter building), and instar age. Moreover, adult performance costs clearly reflected the quality of larval diet, linking early PM exposure to later-life consequences. The responses we observed underline how species- and stage-specific traits, combined with variable PM and TE deposition along roads, directly affect insect performance and contribute to the degradation of roadside ecosystems.

## Methods

### Study organisms

The study focused on two woody plants commonly found in urban greenery and roadside vegetation: *Crataegus monogyna* Jacq. (common hawthorn) and *Prunus cerasifera* Ehrh. (cherry plum, myrobalan plum) (Rosales: Rosaceae). Both host plant species were identified in situ by Dr A. Łukowski (last author), a professional forester with long-term field experience, using the standard identification key^47^. Sampling involved collecting a small number of leaves per tree and was non-destructive, causing no damage to the plants. All sampling was conducted in publicly accessible urban space and did not involve protected species or collection within protected areas. The study complied with applicable institutional and national regulations governing non-destructive sampling of plant material in public space. The sampled trees are long-lived and publicly accessible; if needed, additional leaf material can be re-sampled from the same individuals for independent verification. These species were selected due to their high tolerance to roadside stress conditions typical of urban environments, as well as their ecological importance as host plants for numerous herbivorous insects.

**Fig. 7:**
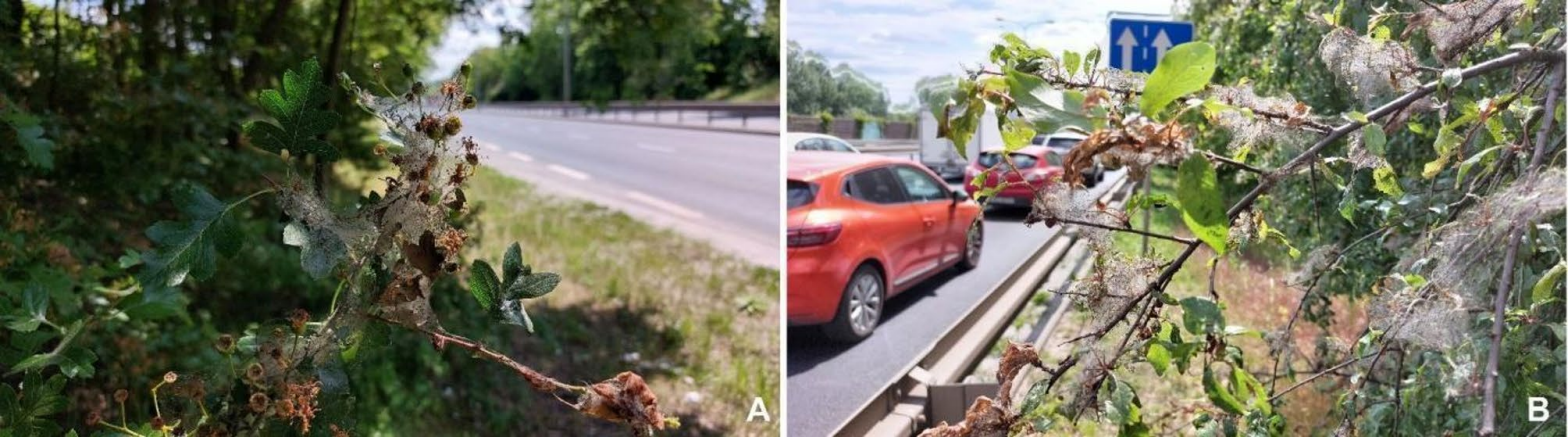
Remains of *Y. padella* larval silken webs on host plants bordering the motorway: A – *C. monogyna*, B – *P. cerasifera*; both represent the highest [R] category of the pollution intensity.

The larvae of *Yponomeuta padella* L. (Lepidoptera: Yponomeutidae) were used as a model organism to evaluate the nutritional quality of the leaves and insect biological responses. *Yponomeuta padella* is classified as a pest species and is included on the list of harmful insects established by the Forest Research Institute in Poland. The species is not legally protected and does not require permits for collection or experimental use. This oligophagous species is strictly dependent on the chemical and physical properties of its host plant’s foliage during larval development. The larvae create ‘tent-like’ nests made of silk and leaves, inside which they feed gregariously and frequently cause complete defoliation of host plants as they move along branches. Consequently, *Y. padella* is not constrained by competition in selecting leaves by other herbivores. Moreover, the nests serve a protective function against predators and parasitoids, and potentially against the direct effects of environmental conditions (Fig. 7).

### Study site and sampling design

The study plot was located in the northern part of Poznań (western Poland), in the vicinity of Lechicka Street (52.4333665°N, 16.9584921°E), within a park adjacent to a major and heavily trafficked urban expressway (National Road no. 92, Poznań, Poland), characterized by substantial vehicular flow, including heavy-duty trucks (see Supplementary Fig. S1 online). The site was chosen to capture a pollution gradient and assess its influence on the leaf traits of the host plants. Plant material was collected across three spatial variants 10 biological replicates (each biological replicate consisted of three technical replicates) for site variant per plant species), differing in degree of exposure to roadside emissions:

- Roadside [R] – leaves and larvae were collected from trees located 1–2 m from the road edge, on the side directly facing the traffic. These trees were subjected to the highest intensity of exhaust fumes, PM, and noise pollution levels.
- Sidewalk [S] – leaves and larvae were collected from the trees and shrubs from the side facing the sidewalk, partially shielded by a vegetation barrier. Although still located within the roadside strip, these samples were less exposed to direct traffic emissions.
- Control [C] – leaves and larvae were collected from plants located behind a 5-meter-tall acoustic barrier separating the road from a neighbouring urban park. Control trees grew at the forest edge. The site featured a stable microclimate and significantly lower exposure to traffic-related stressors.

Nests (*n* = 10 for site variant per plant species) with larvae inside were selected with a preference for nests of similar size and larval density to ensure comparability among replicates, and transported in 1-litre containers, with perforated lids for ventilation, labelled by host species and site (R, S or C), to the laboratory. All samples (leaves and final-instar larvae) were collected simultaneously under uniform weather conditions. Habitat conditions across sites were comparable in terms of associated vegetation composition, vertical structure, and light exposure.

### Sampling and sample preparation

Leaves of *C. monogyna* and *P. cerasifera* were collected during full leaf expansion (May 26, 2023), which represented the optimal phenological stage for assessing their nutritional value for folivorous insects. Collections of foliage were carried out manually, from the lower to mid-canopy layer, in the morning during dry weather to avoid washing off PM. Throughout May, total precipitation amounted to only about 25 mm, with no rainfall recorded during the 10 days preceding the collection, which favoured the accumulation of pollutants.

Plant material was then transported in cooling containers to the laboratory, where it was sorted and processed for further analyses. Leaves were visually screened for mechanical damage and disease symptoms, which were a basis for exclusion, then divided into sub-samples for the following assessments:

- Physical traits (SLA, leaf toughness),
- PM concentration (surface PM, in-wax PM, different PM size fractions, total PM).
- Trace element concentration,
- Biological assays using larvae (choice test).

From the day of larval collection onwards, fresh leaves were delivered daily until pupation began. Strict feeding consistency was maintained: each larval group received only leaves from the same plant and site as their origin.

### Physicochemical leaf analyses

#### Physical parameters

Specific leaf area (SLA), defined as the leaf area per unit dry mass [cm²/mg d.m.], was determined for 180 fresh leaf samples. Leaves were scanned at 300 dpi using a flatbed Canon scanner, and the area was calculated with WinFolia 2016 software (Regent Instruments Inc., USA). Leaves were then oven-dried at 60 °C to constant weight and weighed on an analytical balance (Radwag; accuracy: 0.01 mg). SLA was calculated as the area-to-dry-mass ratio for each leaf.

Leaf toughness was assessed using a penetrometry method with a force gauge (FHT 05), measuring the force (gf/mm²) needed to puncture the leaf blade. Measurements were taken in the central part of the lamina (avoiding the midrib) on every third randomly selected leaf per tree and site (total: 180 leaves; 30 per site nested in the species).

#### Particulate matter on vegetation

Leaf samples (10 biological replicates (each biological replicate consisted of three technical replicates) for site variant per species) were analyzed for PM concentrations using a filtration–gravimetric method developed by Dzierżanowski et al.^12^. The method was designed to assess the effectiveness of vegetation in accumulating airborne PM of different size fractions—large (PM₁₀–₁₀₀), coarse (PM₂.₅–₁₀), and fine (PM₀.₂–₂.₅)—as well as total PM (large + coarse + fine), and in two categories: surface PM (_S_PM) and PM embedded in epicuticular waxes (_W_PM). This method has since been adapted for research on the effects of PM on arthropods, including the ingestion of PM-contaminated foliage, development in PM-polluted habitats, and PM accumulation on insects^9,10,14,48^. Concentrations of PM are given in µg of PM per cm² of leaf area.

#### Trace elements content in foliage

Concentrations of TEs on *C. monogyna* and *P. cerasifera* foliage (10 biological replicates (each biological replicate consisted of three technical replicates) for site variant per species) were quantified with an X-ray fluorescence spectrometry (XRF) analyser Vanta Vac 3 C Series (Olympus, Tokyo, Japan). Prior to measurements, leaf samples were dried and ground in a grinder equipped with a pressure clasp closure and reaching a maximum speed of 32,000 RPM and a 70 μm grinding fineness (Chemland, Poland). Then, the plant material was put into plastic caps A068−100,154 (Traydon) covered with Prolene^®^ Thin-Film (Chemplex Industries Inc., USA). For the elements As, Cd, and Pb, no statistical tests were performed due to the lack of fulfillment of the assumptions of analysis of variance (insufficient number of values, high data variability, lack of data for a specific treatment). The results are presented only in the form of box plots.

### Biological experiments with *Yponomeuta padella*

#### Feeding preference test (choice test)

A three-choice feeding test was conducted using 100 experimental arenas lined with moistened filter paper. Each arena consisted of a plastic Petri dish (⌀ 8 cm) with three side openings (1.5 cm wide, 1 cm high), evenly spaced. Each opening was connected to an external Petri dish, serving as a compartment for a leaf from one of the three site variants (R, S, C). Leaves were obtained from the same plants and kept cool before use.

One fresh leaf per site variant (R, S and C) nested in plant species (*C. monogyna* (*n* = 150 leaves in total; 50 per site variant) or *P. cerasifera* (*n* = 150 leaves in total; 50 per site variant)) was selected per arena and matched for size and maturity. For each plant species, 50 arenas were prepared, ensuring that each site variant was equally represented (see Supplementary Fig. S2 online). After a 4-hour starvation period, a single larva (*n* = 50 per plant species) collected from the control site was placed in the central chamber. The test lasted 24 h under constant conditions (21 °C, 50% RH, LED lighting: 600 lm, 4300 K). A choice was recorded when the larva moved to a leaf and began feeding. No instances of ‘no choice’ were observed.

The initial schematic concept of the experimental setup was generated with the assistance of an AI-based image generation tool (Google Gemini) and subsequently modified, refined, and finalized by the authors.

#### Survival rate, adult emergence dynamic, and body mass

Final-instar *Y. padella* larvae collected from R, S, and C sites were transferred to and kept under laboratory conditions (21 °C, 50% RH, 16 L:8D photoperiod; LED lighting: 600 lm, 4300 K). Late-stage collection allowed for maximum time in natural conditions while enabling controlled pupation and adult emergence. Fresh leaves from the same plant and site were provided daily. Larvae developed undisturbed within their silk structures, mimicking natural conditions.

Daily observations were made to record pupation and adult emergence dates. Larvae spontaneously formed cocoons within ~ 2 weeks. Post-pupation, containers were monitored for emergence success. The number of cocoons, dead larvae and successfully emerged adults was recorded in each container, allowing calculation of imaginal survival. Emerging adults were collected daily, sexed, euthanized with ethyl acetate vapour, and weighed using an analytical microbalance with an accuracy of 0.01 mg (Radwag, Poland).

For each site and host plant species, data were collected on: adult emergence dynamics (time from first to last emergence); number of cocoons, dead larvae and survival success rate (%); adult body mass (separately for males and females).

#### Statistical analysis

Models fit was assessed using the coefficient of determination (R²), adjusted R², and residual diagnostics. All statistical analyses were performed using standard procedures for general linear models. All data were statistically analyzed using the R language (R Core Team version 4.4.2)^49^. Figures were prepared using JMP^®^ (version 19, JMP Statistical Discovery LLC)^50^, and final figure refinement and vector graphic post-processing were performed using Inkscape (version 1.4)^51^.

Chi-square tests were used to analyze the leaf preferences of the larvae. For each species (*P. cerasifera* and *C. monogyna*), a chi-square goodness-of-fit test was performed to check whether the distribution of choices differed significantly from a uniform distribution (33.33% for each site treatment). Additionally, a chi-square test of independence was performed to examine whether there were differences in leaf preferences between species. The level of statistical significance was set at α = 0.05. The results were presented graphically in the form of a mosaic plot showing the percentage share of choices of individual leaf variants for each species.

The emergence dynamics of adult insects from pupae were analyzed using a three-parameter logistic model, which effectively captures the sigmoidal nature of many biological processes. The analysis was based on cumulative percentage data representing the proportion of individuals reaching the adult stage over time. The model was fitted separately for each experimental series (i.e. for each larval nest) with 10 nests per site variant (*n* = 30 per species, totaling 60 for both species). Three key parameters were estimated but only the first two were used for further analysis: the growth rate (reflecting the steepness of the curve), the inflection point (representing the time of the most rapid change, corresponding to 50% of the asymptotic value), and the asymptote. These parameters were used to assess the timing and rate of emergence and to compare dynamics across experimental variants. Model fit was evaluated using the coefficient of determination (R²), residual diagnostics, and information criteria (AIC).

To evaluate the effects of plant species and collection site, a two-way analysis of variance (ANOVA) was used separately for SLA (cm²/g) and leaf toughness (gf/mm²), total PM (µg/cm^2^), sPM (µg/cm^2^), wPM (µg/cm^2^), PM size fractions (µg/cm^2^), TEs content in leaves of the examined host plants (mg/kg), the growth rate (1/day), the inflection point (day), female mass (mg) and male mass (mg). The model included species, site (roadside, sidewalk, control), and their interaction as fixed effects. Individual trees were treated as a random factor to account for within-group variability not explained by the main effects, and when multiple laboratory measurements were obtained per tree, they were averaged so that each tree served as a single biological replicate in the analysis. For each parameter, the coefficient of determination (R²) was reported to indicate the proportion of variance explained by the model.

The percentage of adult emergence (eclosion) was transformed using the Bliss method (arcsine square root transformation of proportions) to meet the assumptions of normality and homoscedasticity. The transformed data were analyzed using a two-way ANOVA, with species (*C. monogyna* and *P. cerasifera*) and collection site (roadside, sidewalk, control) as fixed effects. Interaction effects between species and sites were also tested.

## Supplementary Information

Below is the link to the electronic supplementary material.

Supplementary Material 1

## Data Availability

The data presented in this study are available on Zenodo.org at [https://doi.org/10.5281/zenodo.17071188], reference number 17071188.
