# Supplementary material for: Responses of tree defoliators to traffic-derived particulate matter and trace elements along a roadside pollution gradient
Source: Sci Rep. 2026 Feb 21;16:10069. doi: 10.1038/s41598-026-41296-7 (PMC13021926; doi:10.1038/s41598-026-41296-7)
Supplement: Supplementary file 1 — Supplementary Material 1 [file 41598_2026_41296_MOESM1_ESM.docx]

**Supplementary Information**

**Responses of tree defoliators to traffic-derived particulate matter and trace elements along a roadside pollution gradient**

Hanna Moniuszko*,* Robert Popek, Arkadiusz Przybysz, and Adrian Łukowski


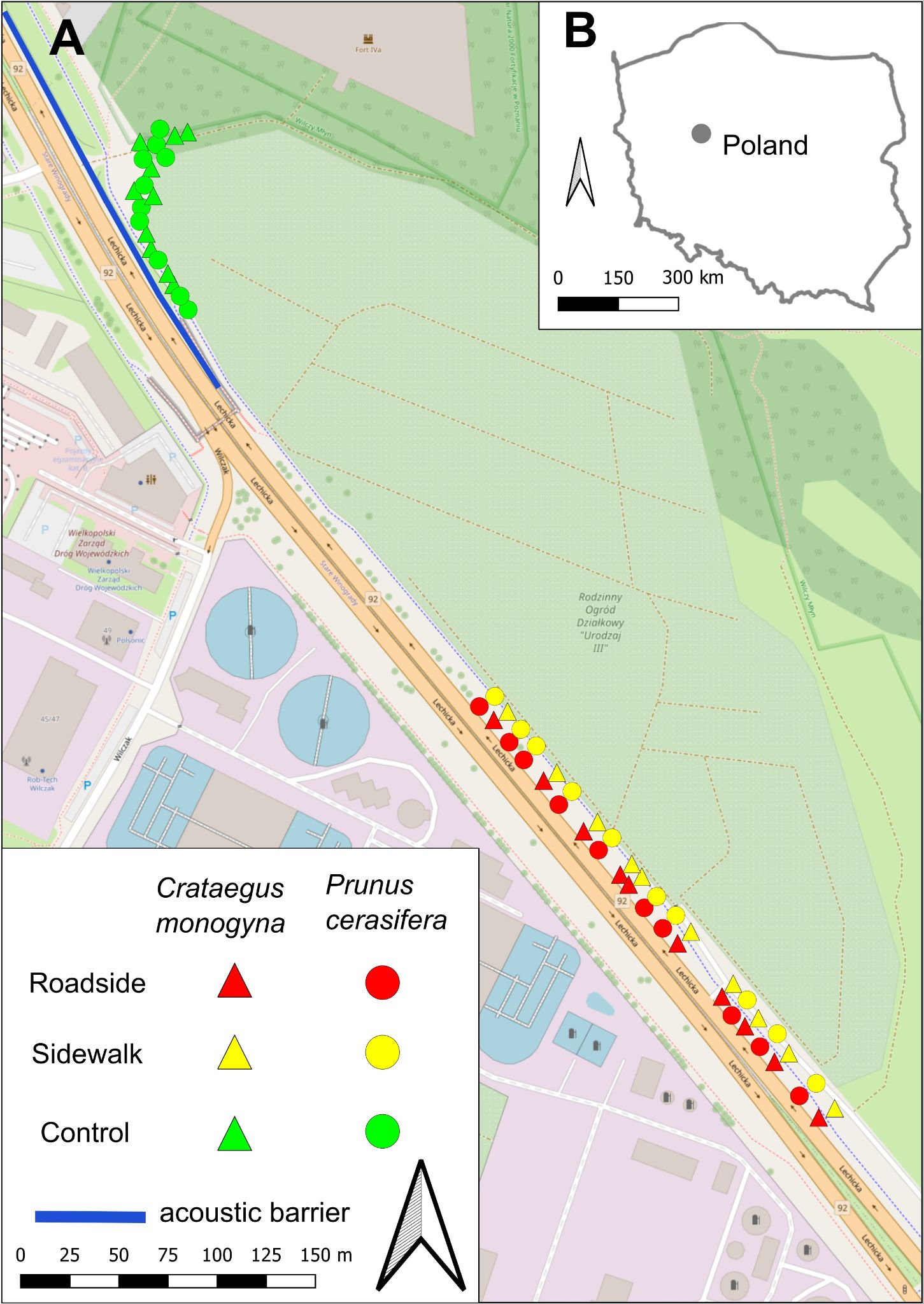


**Supplementary Figure S1.** Location of the study area and sampling design. A: Detailed map of the sampling area along a roadside green belt adjacent to a high-traffic road. Sampling sites were classified into three site variants: roadside (R), sidewalk (S), and control (C). B: Geographic location of the study site within Poland. Symbols indicate sampling locations of *Crataegus monogyna* and *Prunus cerasifera*. After: OpenStreetMap data.


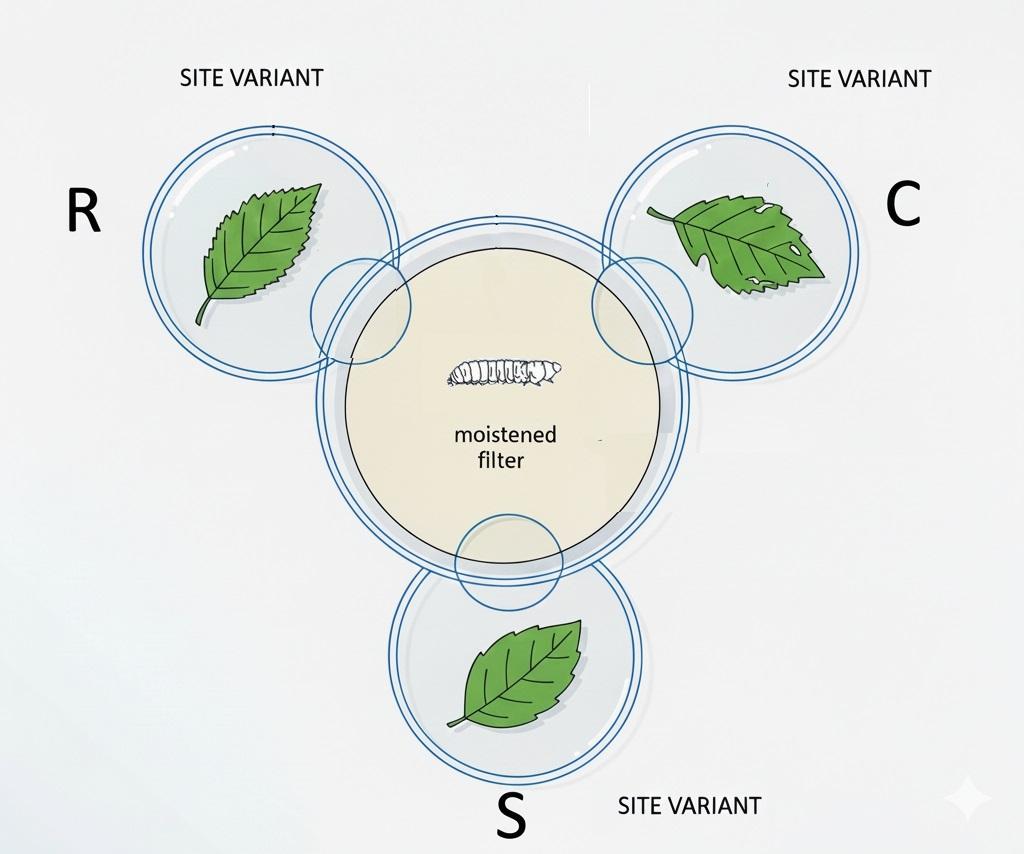


**Supplementary Figure S2.** Schematic representation of the experimental setup used for the choice test. Leaves collected from three site variants—roadside (R), sidewalk (S), and control (C)—were placed equidistantly around a central arena containing a moistened filter. Larvae (n=50 per plant species) were introduced singly into the central arena and allowed to move freely toward the different leaf types.
